# PB.09: Clinical audit of technical recall data for blur following the introduction of the breath hold technique in breast screening

**DOI:** 10.1186/bcr3510

**Published:** 2013-11-08

**Authors:** LE Bisset

**Affiliations:** 1 Dorset Breast Screening Unit, Poole, UK

## Introduction

Digital mammography has increased the number of technical recall (TR) appointments due to blurred images. Within a regional breast screening unit, 177/29,314 women (0.6%) were recalled in an analogue 12-month period compared with 639/30,102 (2.12%) with digital. Current NHSBSP TR standard = 3%, target = 2% [1]. A retrospective audit to assess breath-hold technique TR data aimed to: measure and record data for the TR's pre and post breath hold; compare these results with NHSBSP standards; and make recommendations for future practice based on these results.

## Methods

Datasets gathered information that included mammographic view, radiographer, side of blur, compressed breast thickness, force and location. The data for the pre breath hold sample (8,467) and post breath hold sample (9,072) were compared. A retrospective questionnaire of mammographers' perceptions demonstrated the technique was easy and rarely added additional time.

## Results

Pre breath hold there were 104/8,467 recalls for blurring, and post breath hold there were 31/9,072. The results demonstrate recalls measured against the NHSBSP targets, and TRs dropped from 3.21% (above recommended practice) to 2.08% (in line with standard/almost target recommendations). This is a 69.1% reduction. Fisher's exact test and Pearson's chi-squared with Yates' continuity both produced *P *< 2.2 × 10^-16^. Both were therefore statistically significant for blur.

## Conclusion

The breath hold technique has reduced the number of TRs for blur. Therefore, it is recommended that this technique should be adopted across the entire NHSBSP.
